# Death after Vaccination

**Published:** 1888-10

**Authors:** William Young

**Affiliations:** Atlantic-road, Brixton


### DEATH AFTER VACCINATION.

The following letter was written to the editor of the *Hertfordshire Standard*, England :

DEAR SIR :—The death of Dr. Warren S. Stokes, of Boston, from blood-poisoning caused by vaccination, has produced a profound impression in that city, and in the opinion of a member of the Massachusetts Legislature the compulsory law is doomed, and efforts will be made for its repeal in the next General Court.

From the *Boston Globe* of June 22d, it appears that Dr. Stokes being thrown constantly into contact with infectious diseases, and not having been vaccinated since his early childhood, determined to be re-vaccinated and thus make himself doubly sure against danger. The operation was performed by Dr. Wallace, on Saturday, June 2d. On the following Monday he suffered terribly from nausea and other symptoms of poisoning. Gradually getting worse he was taken on the 9th to the city hospital, where he became delirious, death ending his agonies on the 18th. A post-mortem examination revealed the fact that his death was solely due to blood-poisoning.

Commenting on this sad case the *Banner of Light*, June 30th, says : "As regards the practice of vaccination, we feel no hesitation in declaring it to be our firm conviction that it is a grievous and uumitigated evil, and we earnestly hope that the profound excitement on this subject in Boston will, by stimulating to action those who up to the present time have allowed the disgusting but legally prescribed process to go on unchallenged, bring forth good fruit in an earnest, well-digested, and united effort on the part of her enlightened citizens to sweep away the Compulsory Vaccination Law which now encumbers and disgraces the statute book of the State."

William Young.

Atlantic-road, Brixton, July 21st, 1888.

